# Integrated microRNA and mRNA gene expression in peripheral blood mononuclear cells in response to acute psychosocial stress: a repeated-measures within-subject pilot study

**DOI:** 10.1186/s13104-021-05635-3

**Published:** 2021-06-03

**Authors:** Magdalena Maria Jurkiewicz, Anett Mueller-Alcazar, Dirk Alexander Moser, Indralatha Jayatilaka, Anatoly Mikhailik, Jamie Ferri, Nia Fogelman, Turhan Canli

**Affiliations:** 1 Department of Psychology, Stony Brook University, Stony Brook, NY USA; 2 Program in Neuroscience, Stony Brook University, Stony Brook, NY USA; 3 Program in Genetics, Stony Brook University, Stony Brook, NY USA; 4 Medical Scientist Training Program, Stony Brook University, Stony Brook, NY USA; 5 Deptartment of Molecular Genetics and Microbiology, Stony Brook University, Stony Brook, NY USA; 6 Present Address: Personalized Genomic Medicine/Department of Pathology and Cell Biology, Columbia University Irving Medical Center, New York, NY USA; 7 Present Address: Department of Psychology, MSH Medical School Hamburg, University of Applied Science and Medical University, 20457 Hamburg, Germany; 8 Present Address: Department of Genetic Psychology, Faculty of Psychology, Ruhr-University Bochum, Bochum, Germany; 9 Present Address: Marshall Medical Center, Placerville, CA USA; 10 Present Address: Department of Psychiatry, Yale University, New Haven, CT USA

**Keywords:** Psychosocial stress, Gene expression, microRNA, mRNA, Stress, Inflammation

## Abstract

**Objective:**

The impact of psychosocial stress on a variety of negative health outcomes is well documented, with current research efforts directed at possible mechanisms. Here, we focused on a potential mechanism involving differential expression of mRNA and microRNA in response to acute psychosocial stress. We utilized a validated behavioral paradigm, the Trier Social Stress Test (TSST), to induce acute psychosocial stress in a cohort of volunteers. Stress reactivity was assessed repeatedly during the TSST using saliva samples that were analyzed for levels of cortisol. Peripheral blood mononuclear cells were extracted from blood drawn at baseline and at two time points following the stress paradigm. Total RNA was extracted, and mRNA and microRNA microarrays were utilized to assess within-subject changes in gene expression between baseline and the two post-stressor time points.

**Results:**

For microarray gene expression analysis, we focused on 12 participants who showed a robust cortisol response to the task, as an indicator of robust HPA-axis activation. We discovered a set of mRNAs and miRNAs that exhibited dynamic expression change in response to the TSST in peripheral blood mononuclear cells, further characterizing the link between psychosocial stress and cellular response mechanisms.

**Supplementary Information:**

The online version contains supplementary material available at 10.1186/s13104-021-05635-3.

## Introduction

Organismal stress responses are associated with widespread changes in the transcriptome of circulating peripheral blood mononuclear cells (PBMCs), with changes in expression of genes regulating cell signaling, adhesion, differentiation, and immune pathways [1]. Such stress-associated transcriptional changes in PBMCs are mediated, in part, through microRNAs (miRNA) [2, 3], such as miRs 7b, 16, 20b, 21, 26b, 29a, 29c, 126 and 144 [4–7].

A popular laboratory-based paradigm for the study of psychosocial stress physiology is the Trier Social Stress Test (TSST) [8]. A previous study reported TSST-associated changes in salivary levels of miR-20b, -21 and 26b [9], but their expression levels in PBMCs and their association with gene expression remain unknown. Here, we report results from a TSST pilot study examining within-subject repeated-measures of mRNA and miRNA in PBMCs.

## Main text

### Methods

#### Participants

Participants were Caucasian males recruited via flyers posted on the Stony Brook University campus and through advertisements in local newspapers in surrounding communities on Long Island, NY, USA. Study exclusion criteria included: age under 18 years, significant current or prior presence of psychiatric illness, smoking, presence of substance or alcohol abuse in the past six months, hormonal, mood-altering, heart disease and/or cardiovascular conditions related, or psychoactive drug medication, BMI ≥ 30. Participants were pre-screened for exclusion criteria by phone. Fifty-five participants were part of the study, which was approved by the Institutional Review Board (IRB) and Committee on Research Involving Human Subjects (CORIHS) of Stony Brook University, Stony Brook, USA. We selected 12 participants who showed a robust cortisol response to the TSST (“Cortisol Responders”; based on a criterion suggested by Miller et al. [10]) as a discovery cohort to generate repeated-measures, within-subject genome-wide mRNA and miRNA expression data.

#### Psychosocial stress procedure: TSST

All participants were scheduled for a 4-h weekday laboratory session conducted at the General Clinical Research Center (GCRC) at the Stony Brook University Hospital from 1400 to 1800 h. Upon arrival, participants were informed about the study, completed the consent procedure, and underwent insertion of an intravenous catheter into the arm for the first blood draw. Participants then rested for a period of at least 45 min before they were introduced to the psychosocial stress paradigm. The Trier Social Stress Test (TSST) is a standardized social stress protocol that was used to induce psychosocial stress and that has been found to elicit a strong and reliable physiological response to laboratory stress [8]. Briefly, the standardized laboratory stressor consists of a 5-min preparation period, a 5-min free speech task during which participants give a mock job interview, and a 5-min mental arithmetic task wherein participants perform serial subtraction by seventeen all in front of a non-responsive audience.

#### Biological measures: cortisol

To assess cortisol levels, ten salivary samples were obtained from each participant throughout the duration of the study. Samples were collected using Salivettes^®^ (Sarstedt) at arrival, 2 min before beginning the TSST, and again at 2, 10, 20, 30, 45, 60, 90- and 105-min post stress task. Participants were instructed to gently chew on the cotton swab for approximately one minute during each collection. Cotton swabs were then transferred to plastic containers and immediately stored at − 20 °C until shipment to the analyzing laboratory (N. Rohleder, Brandeis University, Waltham, MA, USA). Salivary cortisol concentrations were measured using a commercially available chemiluminescence immunoassay (CLIA; RE62019; IBL International, Toronto, Canada). Inter-assay variability was 3.3%, and intra-assay variability was 3.4%. Salivary cortisol concentrations have been shown to highly correlate with serum cortisol concentrations, making salivary cortisol a reliable indicator of HPA activity [11]. Participants had no oral intake except for water for 3 h before beginning the study session. Cortisol response to the stress paradigm was assessed by subtracting the highest mean cortisol concentration following the stressor minus the baseline cortisol concentration (two minutes before the TSST). Using a criterion suggested by Miller et al. [10], those participants who showed a cortisol increase of at least 1.5 nmol/l from baseline were assigned in the “Cortisol Responder” group (n = 33, mean cortisol increase of 8.55 nmol/l (± 5.63), while those who did not show an increase of at least 1.5 nmol/l from baseline were placed in the “Cortisol Non-Responder” group (N = 22, mean cortisol increase of − 3.889 nmol/l (± 6.45). The two groups differed in age (responders: 34.58 years ± 17.09; non-responders: 26.18 years ± 13.44; t-test *p* < 0.05). There were no significant differences in psychological variables of interest, as the BAI, BDI, TiCS, STAI-S and CTQ sum scores were not significantly different between the two groups (all *p* > 0.05). There was no significant difference in the number of stressful life events (SLEs) between the two groups (*p* > 0.05). Subsequent gene expression analyses with microarray profiling were conducted on the first 12 Cortisol Responders.

#### Biological measures: blood collection

Directly upon arrival and following the informed consent procedure, an intravenous (IV) catheter was inserted into each participant’s arm for the first blood draw (Time Point A). Participants then rested for a period of at least 45 min before they were introduced to the psychosocial stress paradigm. The second blood draw occurred 45 min after the stress paradigm (Time Point B) and the third blood draw occurred 105 min post stress (Time Point C). Ten ml of EDTA blood (Vacuette^R^ Greiner Bio-one) was collected at each of the three time points. Blood was immediately processed for PBMC extraction according to the Leucosep^®^ Instruction Manual (Greiner Bio-one), and cell pellets were frozen and stored at − 80 °C.

#### RNA isolation, quantification, quality control

Total RNA was extracted from PBMCs at each time point using the Qiagen All-Prep kit with company-recommended modification to extract small RNA (Qiagen). RNA quantity was assessed using a Nanodrop Technologies ND-1000 instrument (NanoDrop Technologies). Total RNA quality was measured using the Agilent RNA 6000 Pico kit and the Agilent Bioanalyzer 2100A (Agilent Technologies), while miRNA content was resolved using the Small RNA Kit (Agilent). RNA samples were stored at − 80 °C.

#### MiRNA microarray profiling

miRNA microarray profiling was performed at the Microarray Core Facility of Cold Spring Harbor Laboratory using the Affymetrix GeneChip miRNA 2.0 Array according to the manufacturer’s protocol. Briefly, 400 ng of total RNA was labelled using the 3DNA Array Detection FlashTag™ Biotin HSR Kit following the manufacturer's recommendations (Genisphere). Arrays were scanned on the Affymetrix GeneChip Scanner 3000, and feature extraction was conducted using Affymetrix Command Console software. Microarray background correction, quantile normalization, probe set summarization, and log2 transformation were performed with the miRNA QC Tool Version 1.1.1.0 (Affymetrix).

#### MRNA microarray profiling

mRNA microarray profiling was performed at the ShanghaiBio Corporation using Affymetrix Human Gene ST 1.0 Arrays according to the manufacturer’s protocol. Briefly, 500 ng of total RNA was used in complementary DNA synthesis and labeling, followed by hybridization, washing, scanning, and image processing of GeneChips according to standard Affymetrix protocols. Quantitative image files were loaded into the Bioconductor Affymetrix linear modeling Graphical User Interface software [12] and background adjustment, quantile normalization, probe summarization, and log2 transformation was performed using the Robust Multi-Array Average method [13].

#### Statistical analysis of mRNA and miRNA microarray data

Time-course microarray data analysis was conducted using the Linear Models for Microarray Data (limma) Bioconductor package [14]. Linear models were fitted to distinguish differentially expressed mRNAs between the three time points (A, B, C) on the Affymetrix platform. mRNA showing limma *p* < 0.01 for at least one of the time point comparisons (B to A, C to A, B to C) correcting for multiple comparisons using false discovery rate (FDR) correction, as well as with a fold change (FC) |FC|≥ 1.3 were highlighted as genes that show greater variation in expression in response to the TSST. The fold change threshold of |FC|≥ 1.3 was chosen as literature has conventionally utilized fold changes ≥ 1.3–2 to suggest differential expression in mRNA microarray data [15]. Also, fold change of gene expression in blood has usually been less than two-fold [3, 16] and multiple studies have used a cut-off of FC > 1.2 for identifying changes of gene expression specifically in blood [16–18]. miRNAs generally show more subtle changes in expression than mRNAs, but even small expression changes can have significant biological effect as each miRNA has multiple gene targets and can influence multiple genes in a given pathway. Therefore, miRNAs showing a |FC|≥ 1.2 and limma *p* value < 0.01 for at least one of the time point comparisons (B to A, C to A, B to C) were highlighted as showing change in expression in response to the TSST.

#### MiRNA/mRNA integration and functional profiling

Integration of mRNA and miRNA expression was performed by the computational gene network prediction tool Ingenuity Pathway Analysis (IPA, Ingenuity^®^ Systems). Our study showed a limited number of mRNAs that were significant according to limma p < 0.01 and |FC|≥ 1.3, possibly in part due to the small study sample size and conservative statistical inclusion criteria. It is worthwhile noting that a single miRNA can subtly regulate multiple genes in a given pathway or process, having an additive biological effect. Therefore, mRNAs that showed significant changes in expression at any time contrast according to limma p < 0.01 were input into IPA. In parallel, human orthologues of differentially expressed miRNAs (limma p < 0.01 and |FC|≥ 1.2) were input into the IPA program as well. To integrate the two datasets, identification of negatively correlated miRNA/mRNA pairs was performed using the Target Filter Analysis in IPA. The algorithm uses experimentally validated interactions as well as predicted microRNA–mRNA interactions from TargetScan. Fold change at each miRNA and mRNA was integrated at Time B vs Time A, Time C vs Time A, and Time C vs Time B. The expression pairing analysis was used to identify negatively correlated miRNA/mRNA pairs that had experimentally demonstrated targeting or a moderate to high degree of likelihood of a targeting relationship based on prediction algorithms.

## Results

### Participant characteristics and gene expression analysis

Table 1 lists participant characteristics and Table 2 lists 9 mRNAs that were differentially expressed according to the criteria of limma p < 0.01 and |FC|≥ 1.3. mRNAs that showed significant changes in expression at any time contrast according to limma p < 0.01 were put into IPA for expression pairing and are presented in Additional file 1: Table S1. Limma analysis revealed 33 miRNA probe sets on the Affymetrix array that showed significant expression changes (p < 0.01 and |FC|> 1.2) in Cortisol Responders over the course of the TSST. Only the miRNAs with identified human orthologues that readily mapped into IPA were considered in further analysis. In the instance that stem-loops were identified by the microarray, both mature products were put into IPA when as per verbal instructions from IPA Technical Support. The list of resulting miRNAs is presented in Table 3.

**Table 1:** TSST participant characteristics

Sample	Cortisol responders profiled by microarray
N	12
Age (years)	43.17 (19.34)
Cortisol response (in nmol/l)	13.60 (5.77)
BDI	2.36 (2.38)
BAI	3.75 (3.05)
TiCS-CSSS	10.33 (7.24)
CTQ	40.90 (14.62)
STAI-S	25.00 (5.82)
SLE	45.75 (11.08)

All values are mean (± standard deviation). Higher sum scores indicate increased symptom severity

*BAI* beck anxiety questionnaire, sum score, *BDI* beck depression questionnaire, sum score, *TiCS-CSSS* chronic stress screening scale of the trier inventory for the assessment of chronic stress, sum score, *CTQ* childhood trauma questionnaire, sum score, *STAI* state-trait anxiety questionnaire, *SLE* stressful life events (based on a structured interview)

**Table 2:** Differentially expressed mRNA transcripts in cortisol responders

Symbol	Gene name	Fold change		
		B vs A	C vs A	C vs B
RNU6-82P	RNA, U6 small nuclear 82, pseudogene	**1.5**	1.2	− 1.2
ZRANB2	Zinc finger, RAN-binding domain containing 2	**1.4**	1.2	− 1.2
SPON2	Spondin 2, extracellular matrix protein	**− 1.3**	**− 1.4**	− 1.0
AKR1C3	Aldo–keto reductase family 1, member C3	− 1.3	**− 1.4**	− 1.1
GOLGA8DP	Golgin A8 family, member D, pseudogene	− 1.1	**− 1.3**	− 1.2
TCF19	Transcription factor 19	− 1.1	**− 1.3**	− 1.2
RPS9	Ribosomal protein S9	1.0	1.4	**1.5**
TGIF2LX	TGFB-induced factor homeobox 2-like, X-linked	1.1	− 1.2	**− 1.3**
RNF144B	Ring finger protein 144B	1.3	− 1.0	**− 1.3**

This table presents differentially expressed mRNAs in cortisol responders according to the criteria limma (p < 0.01 and |FC|≥ 1.3) over the course of the TSST

**Table 3:** Differentially expressed miRNAs in cortisol responders

MIR	Fold-change		
	B vs A	C vs A	C vs B
miR-92a-2	− 1.2	1.0	1.2
miR-129-5p	1.2	1.1	− 1.1
miR-518a-2	− 1.2	1.0	1.2
miR-9-5p	− 1.1	− 1.3	− 1.2
miR-137-3p	− 1.2	− 1.1	1.1
miR-624-3p	− 1.1	1.2	1.3
miR-614	− 1.2	− 1.1	1.2
miR-3178	− 1.2	− 1.2	− 1.0
miR-375-3p	1.1	− 1.1	− 1.2
miR-210-3p	1.0	− 1.3	− 1.3
miR-3191-3p	1.1	− 1.1	− 1.2
miR-1185-5p	1.1	− 1.1	− 1.2
miR-937-3p	1.1	1.2	1.1
miR-301b	1.1	− 1.1	− 1.2

This table presents differentially expressed miRNAs in cortisol responders according to the criteria limma (p < 0.01 and |FC|≥ 1.2) over the course of the TSST. Only miRNAs with identified human orthologues that readily mapped into Ingenuity Pathway Analysis (IPA) were considered. In the instance that stem-loops were identified by the microarray, both mature products were input into IPA

### MiRNA/mRNA integration

Negatively correlated miRNA/mRNA pairs were identified at contrasts B vs A, C vs A, or C vs B using IPA’s Target Filter Analysis. Since miRNAs often result in subtle changes in gene expression, mRNAs that showed significant change in expression according to limma p < 0.01 were used in an expression-pairing analysis along with miRNAs significant according to criteria limma p < 0.01 and |FC|≥ 1.2. Resultant miRNA/mRNA expression pairs at each time contrast are listed in Additional file 2: Table S2A–C.

## Discussion

This pilot study examined genome-wide changes in mRNA and miRNA gene expression in PBMCs in response to TSST-induced psychosocial stress within-subjects in a repeated-measures design. By removing between-subject genomic variance that would otherwise obscure any relationship between stress and gene regulation, we could identify differentially expressed miRs and target mRNAs at FDR-corrected levels, although we replicated none of the previously reported stress-related miRs. Non-replication is common is small-scale studies, but the aggregate of our results and previous reports suggests that properly powered large-scale studies of stress-related gene regulation by miRNAs are warranted to better understand the molecular mechanisms of stress-related gene dysregulation.

## Limitations

Limitations of this study include a small sample size consisting of healthy Caucasian males only. Thus, cautious interpretation is advised. Replication in a larger and more diverse samples that are more broadly representative of the variation in human stress-related phenotypes should be considered in future work.

In addition, microarray results should be confirmed by an orthologous method such as RT-qPCR in order to confirm changes in gene expression.

## Supplementary Information

**Additional file 1:** **Table S1A–C**. Differentially Expressed mRNAs according to limma p < 0.01. These tables list mRNAs found to be differentially expressed according to limma p < 0.01 for one or more of the contrasts (Time Point B vs Time Point A, Time Point C vs Time Point A, and Time Point C vs Time Point B). This is the complete list of genes used in IPA pathway analysis. mRNAs highlighted in yellow have fold change ≥ 1.3.

**Additional file 2: Table S2A–C**. mRNA/miRNA expression pairings. Negatively correlated mRNA/miRNA pairs identified by IPA Target Filter analysis at Time Point B vs A, Time Point C vs A and Time Point C vs B are presented in Table S2A-C, respectively.

## Data Availability

The datasets analyzed during the current study are available from the corresponding author on reasonable request.
